# Rapid serogroup classification of the footrot pathogen *Dichelobacter nodosus* using multiplex qPCR of lesion samples from sheep in the Netherlands

**DOI:** 10.3389/fvets.2025.1683551

**Published:** 2026-01-21

**Authors:** Birgitta Duim, Niels Dekker, Reinard R. Everts, Margit Groenevelt, Joost Hoogeveen, Arjen Timmerman, Heleen Zweerus, Marian J. Broekhuizen-Stins, Mohammad Mokbel, Om P. Dhungyel

**Affiliations:** 1 Department of Biomolecular Health Sciences, Faculty of Veterinary Medicine, Utrecht University, Utrecht, Netherlands; 2 Diergeneeskundig Centrum Zuid-Oost Drenthe, Coevorden, Netherlands; 3 Dutch Sheep and Goat Breeders Association (NSFO), Zaltbommel, Netherlands; 4 Sustainable Ruminant Health, Department of Population Health Sciences, Faculty of Veterinary Medicine, Utrecht University, Utrecht, Netherlands; 5 Farm Animal Health, Sydney School of Veterinary Science, The University of Sydney, Sydney, NSW, Australia

**Keywords:** fimbriae, footrot vaccination, qPCR, serotyping, sheep, *Dichelobacter nodosus*

## Abstract

*Dichelobacter nodosus (D. nodosus)* is the pathogen responsible for causing footrot in sheep and goats, which poses significant challenges to animal health and welfare. *D. nodosus* is classified into 10 different serogroups (A–I and M) based on the genetic variation of this fimbrial (*fimA*) gene. These fimbriae are immunogenic and play an important role in virulence, making serotyping of these fimbriae valuable for identification and vaccine development. In this study, three multiplex quantitative polymerase chain reaction (qPCR) assays, targeting the most commonly prevalent nine serogroups (ABC, DEF, and GHI), were studied for the detection of serogroups in foot swab samples collected from Dutch sheep farms. A total of 147 samples tested positive for *D. nodosus* using *pnpA* qPCR, and 144 (98%) samples exhibited a serogroup using qPCR. The multiplex qPCRs detected significantly more serogroups than conventional serogroup PCRs and detected more than one serogroup in a swab. In 46 samples (31%, 46/147), two to five different serogroups were identified from a single swab sample. In three samples, no serogroup was identified, likely due to sequence variation in the *fimA* gene in these samples. These direct multiplex qPCR tests provide faster, more sensitive, and accurate testing for the direct classification and quantification of *D. nodosus* serogroups for studying the epidemiology of footrot and for the formulation of serogroup-specific targeted vaccination strategies for prevention, control, and treatment of footrot.

## Introduction

*Dichelobacter nodosus* is a fastidious anaerobic bacterium and is the essential causative agent for footrot, the major cause of lameness in sheep and goats (1). Footrot lesions begin as interdigital dermatitis that can progress to necrotic separation of the hoof horn from the underlying tissue (2). The major known immunogens of *D. nodosus* are the fimbriae (pili), the surface (K) antigens, which are responsible for the K-agglutination reaction and form the basis of antigenic variation of serogroups (strains) (3, 4). This antigenic variation resides in the fimbrial proteins and their subunit gene (*fimA*), and this feature has been used to classify isolates into 10 serogroups (A–I and M) (5–7). Depending on the homology of the fimbrial sequence, *D. nodosus* fimbriae have been classified into two classes, namely Class I or A-set fimA1 and Class II or D-set fimA2 (8, 9). Class I consists of serogroups A, B, C, E, F, G, I, and M, and Class II consists of serogroups D and H. The elicited immunity has been shown to be serogroup-specific, and the presence of multiple serogroups is common in the affected flocks of sheep (10–12). A commercial multivalent vaccine containing all the serogroups except M has been proven to provide relatively poor efficacy and effectiveness for individual serogroups due to the phenomenon of antigenic competition (13–15). Higher efficacy and effectiveness in control, prevention, and eradication have been observed using outbreak-specific vaccination with monovalent or bivalent vaccines (16–19). Multiple serogroup infections in a flock can also be successfully controlled and eradicated by sequentially targeting two serogroups with bivalent vaccines, targeting the most prevalent serogroups first and sequentially targeting the remaining serogroups accordingly (16, 20). The basis of this success of outbreak-targeted vaccination lies in reliable and preferably affordable and fast diagnostics.

The approach of outbreak-specific, sequentially targeted vaccination is most successful when the proportion of circulating serogroups is determined, allowing for a sequential administration of bivalent vaccines in order of prevalence. The underlying assumption is that only the dominant serogroups are clinically relevant and associated with disease. Moreover, these infections often occur in conjunction with other foot pathogens, such as *Fusobacterium necrophorum* and *Treponema* spp., indicating a complex polymicrobial etiology (21, 22). Strategic vaccination with bivalent *D. nodosus* vaccines not only helps in disease prevention and control but also contributes to a better understanding of the pathogenesis and microbial interactions involved in ovine foot infections.

Essential for a strategic vaccination approach is the reliable identification of the prevalent *D. nodosus* serogroup(s) in a flock of sheep. The flock-level footrot infection status is typically assessed through clinical examinations, bacterial culture, and traditional PCR-based methods (23, 24). However, bacterial culture methods are fastidious, require specialized media, and are not sensitive enough to detect all the serogroups in a sample (25). Conventional PCR assays (cPCR) currently available targeting the *fimA* gene for serogroup detection are found to be quicker and more sensitive than culture methods (5). However, real-time quantitative PCR (qPCR) offers even greater sensitivity, specificity, and the ability to quantify target DNA. In this study, we describe the development of a sensitive multiplex quantitative PCR (qPCR) for direct detection, classification, and quantification of the *D. nodosus* serogroups in direct swab samples from sheep at farms with a known history of footrot.

## Materials and methods

### Real-time qPCR design

A multiplex qPCR for the detection of nine serogroups of *D. nodosus* classified by sequence variation in the *fimA* gene was designed after alignment of *fimA* sequences using the ClustalW algorithm in MEGA v6.06 software 26. Serogroup M was not investigated due to the low number of sequences for designing a serogroup-specific probe. For the most widely prevalent nine serogroups, a combination of primers and probes was designed to be specific for each serogroup using Primer Express v3.0.1 (ThermoFisher Scientific) and resulted in three sets of multiplex qPCR tests for serogroups ABC, DEF, and GHI.

The amplicon size of the selected qPCR targets was small, and the specificity was increased by adding a minor groove-binding modification (MGB) (Table 1). The *in silico* specificity of all primer and probe sets was checked using the basic local alignment tool BLASTn by accessing the NCBI GenBank containing 183 genomes, from which 95 contained serogroup data.

**Table 1:** *Dichelobacter nodosus* serogroup multiplex qPCR design.

Sero group	F primer (5′-3′)	R primer (5′-3′)	Probe	Fragment size (bp)	Multiplex
A	GAAGGTTCGCATTTCTGATCACT	GCAGCTGGGTTCGCATCTT	FAM-AGAAAGCGGTGAATGTA-MGB-BHQ1	63	1
B	AGAAGCTGGCGATCCGAAT	TTCTGCAGTGCCTTGACCATAA	YY-TTGTAAGGTCGAAATCA- MGB	61	
C	CGCTATCCCTGCATACAACGA	GGATTTTTAAACCATCAGCTAAGCTT	LC610-TACATTGCTCGTACCCAAGTTTCTGAAGGC-BHQ2	80	
D	CGCACCGCCATCGAAA	CCAACCAATGAAGCATTTATCG	FAM-TTGCGTTTTGGATGGTAAA-BHQ1	63	2
E	TCTCCAAGTTGATCGATACCAAAG	CGCCTTGGGTGTAAGAACCA	YY-TTAGAACTTGAACAATTGGT-MGB-BHQ1	69	
F	TATGGAAACTGCTAATGCTGG	GTGATTGTAACTTTACAA	LC610-GGTCTAGCCGAAATCAGT-MGB-BHQ1	138	
G	TCCGTATCGCTGACAACTTACAA	CTTCGCCAGATGCAGGATCT	FAM-ATGGTAAATGTACCTCTGAAG-MGB-BHQ1	66	3
H	GCACTTGAATCCACTGCTGAAA	AAGTGTAGCGGCAGCATTCTG	YY-TAAGATTGAAGCTACATTTGG-MGB-BHQ1	66	
I	GGCGTAAGCTTAGCTGATGGTTT	CCAGTCGATGGGTCAGCAT	LC610-TCCGCATCGCTGAGAACTTGCAAGAC-BHQ2	92	
M	WKCTGGTGAAAAAGGTAACS	TGATCCATAAGTAATAGTTACGAC	FAM-AGCWGTAATCAGTGGTACTTATNATGMGB-BHQ1	127	4
IC-PhHV	GGGCGAATCACAGATTGAATC	GCGGTTCCAAACGTACCAA	Cy-5-TTTTTATGTGTCCGCCACCATCTGGATC-BHQ1	89	

F = forward primer, R = reverse primer, bp = basepair, internal control = IC-PhHV - Phocine Herpes virus.

### Serogroup multiplex qPCR

Each set of PCR serogroup primers for the multiplex qPCR assays was optimized and developed using similar reagent compositions, concentrations, and cycling conditions. Reactions consisted of 20 μL with 10 μL 2x LC480 Probes Master (Roche Diagnostics, Almere, the Netherlands), 5 μL of purified DNA, different volumes of primers and probes (Table 1), and molecular grade water. The qPCR program on the LightCycler LC480 was as follows: an initial incubation of 10 min at 95 °C, followed by 40 cycles of denaturation at 95 °C for 15 s and annealing/extension at 58 °C for 60 s. Samples were considered positive for a serogroup if the LC480 software assigned a Ct-value of <40. No template controls (molecular grade water) or positive controls, consisting of standardized genomic DNA for each Australian prototype serogroup, were included in any of the qPCR runs.

### Analytical sensitivity of the serogroup qPCR using reference strains of *D. nodosus*

The multiplex qPCR was evaluated on genomic DNA from a panel of reference Australian prototype strains of *D. nodosus*, representing serogroups A to I (5). A pure culture of each prototype strain of *D. nodosus* was grown on 4% hoof agar media and harvested with a cotton-tipped swab in 300 μL of phosphate-buffered saline. DNA was extracted using the DNeasy UltraClean Microbial Kit (Qiagen, Veldhoven, the Netherlands) or the Wizard Genomic DNA Purification Kit (Promega, USA). A triplicate 10-fold serial dilution of chromosomal DNA was used to create a standard curve for serogroup quantification.

The serogroup specificity was checked for cross-reactivity between serogroups. DNA of the serogroups was tested in a checkerboard setup with singleplex primer pairs and the multiplex of three serogroups each against single and multiple DNA templates.

### qPCR detection of serogroups of *Dichelobacter nodosus* in swabs

Evaluation of the multiplex qPCRs (ABC, DEF, and GHI) was performed on a set of foot lesion swab samples that were collected for monitoring purposes. All samples included in this evaluation (*n* = 240) were taken from sheep from a total of 24 Dutch flocks with a known history of clinical footrot. As described by McPherson et al. (23), the sampling procedure was performed by veterinarians with the assistance of veterinary students. In each selected sheep, only the foot with the most severe lesion was swabbed. The swabs (CLASSIQSwabs, Copan Italia, Italy) were suspended in 800 μL of lysis (LA) buffer with a PhHV internal control. The samples were centrifuged at 20,000 rpm x for 3 min at room temperature, and 200 μL was used for DNA extraction using a DNeasy Blood and Tissue Kit (Qiagen) (26). The presence/absence of *D. nodosus* was detected using a qPCR targeting the *pnpA* gene (27, 28). The samples that were *pnpA* qPCR-positive (Ct-value <35) were analyzed with the serogroup qPCRs. Negative control samples tested in each qPCR run were nucleic acids purified from *pnpA-*negative swabs. When qPCR of the internal control was higher than the Ct-value of 33, they were diluted at a 1:10 ratio and repeated. When this value did not improve, a sample was considered *D. nodosus* negative (27, 28).

### Conventional serogroup PCR

The performance of the multiplex serogroup qPCR assays (ABC, DEF, and GHI) was evaluated against the cPCR assay for each serogroup (A–I). All cPCR assays were conducted as previously described (6, 23). A positive result for each serogroup cPCR was indicated by the presence of an amplification product of the expected size, visualized after electrophoresis on a 2% agarose gel stained with RedSafe (iNtRON Biotechnology, Republic of Korea), and viewed under ultraviolet light.

### Statistical analysis

The amplification efficiencies of the multiplex qPCR were calculated from the slopes of the standard curves and expressed as Ct-value versus log_10_ DNA concentration using the equation *E* = 10 ^-1/slope^ – 1, expressed as percent efficiency (*E*) and the *R*^2^ correlation coefficient for variation in linearity of the amplification standard curve (Table 2 and Supplementary Table 1). The analytical sensitivity was calculated from the standard curve as the lowest concentration of serogroup-specific *D. nodosus* DNA detectable (LOD). The McNemar’s statistical test was used for comparing the serogroups detected in a sample by qPCR versus cPCR. The Wilcoxon signed-rank test was used for comparison of the total number of serogroups per sample detected by qPCR versus cPCR.

**Table 2:** Analytical sensitivity of the qPCR assays.

Multiplex	1			2			3		
Parameters	A	B	C	D	E	F	G	H	I
*R^2^*	0.999	0.995	0.999	0.999	0.999	0.998	0.996	0.999	1.00
% efficiency (*E*)	94	102	96	99	100	103	105	106	97
LOD pg./μl	0.5	0.1	0.3	0.08	0.25	0.4	0.13	0.13	1.15

Individual serogroup qPCR									
Parameters	A	B	C	D	E	F	G	H	I
*R^2^*	0.999	0.999	0.999	0.999	0.999	0.999	0.999	0.999	0.999
% efficiency	97.8	95	90	101	99	91	97	91	95
LOD pg./μl	0.5	0.1	0.3	0.08	0.25	0.4	0.013	0.013	1.15

## Results

### Analytical sensitivity and specificity

To confirm the specificity of the primers and probes, an *in silico* analysis was conducted using public sequence databases. Primer-BLASTn results indicated that the oligonucleotides were serogroup-specific. The alignment identified suitable combinations of primers and probes for each serogroup, allowing for the development of three multiplex formats, as shown in Table 1.

The limit of detection (LOD) and the amplification efficiency of the *D. nodosus* serogroup-specific qPCR assays showed detection of a maximum of 125 ng, corresponding to 8 × 10^7^ copies, to a minimum of 12.5 fg, corresponding to 8 copies, calculated from the *D. nodosus* VCS1703A reference genome per reaction (Supplementary Table 1). The results indicated that each individual serogroup qPCR, as well as all targets within the multiplex format, demonstrated an amplification efficiency of 90% or greater with a coefficient of correlation of > 0.99. The limit of detection (LOD) for the multiplex qPCRs ranged from 0.01 pg. (8 copies) to 0.08 pg. (55 copies) (Table 2). Notably, for serogroups A and I, the detection sensitivity was found to be 10 times higher in the multiplex qPCR format than running these serogroup qPCRs individually.

The analytical sensitivity of the multiplex qPCR demonstrated an LOD for each serogroup that was generally 10 times higher than that of cPCR assays (data not presented). The serogroup specificity test (Table 3) showed no cross-reactions for the primer pairs or multiplex combinations, except for high Ct-values (cycles >37), indicating cross-reactivity between serogroups G and C.

**Table 3:** Serogroup specificity of single plex primer pairs and multiplex serogroup primers.

Primer combinations	serogroup								
	A	B	C	D	E	F	G	H	I
AF + AR	+	−	−	−	−	−	−	−	−
BF + BR	−	+	−	−	−	−	−	−	−
CF + CR	−	−	+	−	−	−	-*	−	−
DF + DR	−	−	−	+	−	−	−	−	−
EF + ER	−	−	−	−	+	−	−	−	−
FF + FR	−	−	−	−	−	+	−	−	−
GF + GR	−	−	–*	−	−	−	+	−	−
HF + HR	−	−	−	−	−	−	−	+	−
IF + IR	−	−	−	−	−	−	−	−	+
AFAR + BFBR + CFCR	+	+	+	−	−	−	−	−	−
DFDR + EFER + FFFR	−	−	−	+	+	+	−	−	−
GFGR + HFHR + IFIR	−	−	−	−	−	−	+	+	+

F = forward primer, R = reverse primer, A-I = serogroups, *Cross reactions seen at cycles 37 and 38.

### qPCR serogroup detection in clinical swabs

**Figure 1:**
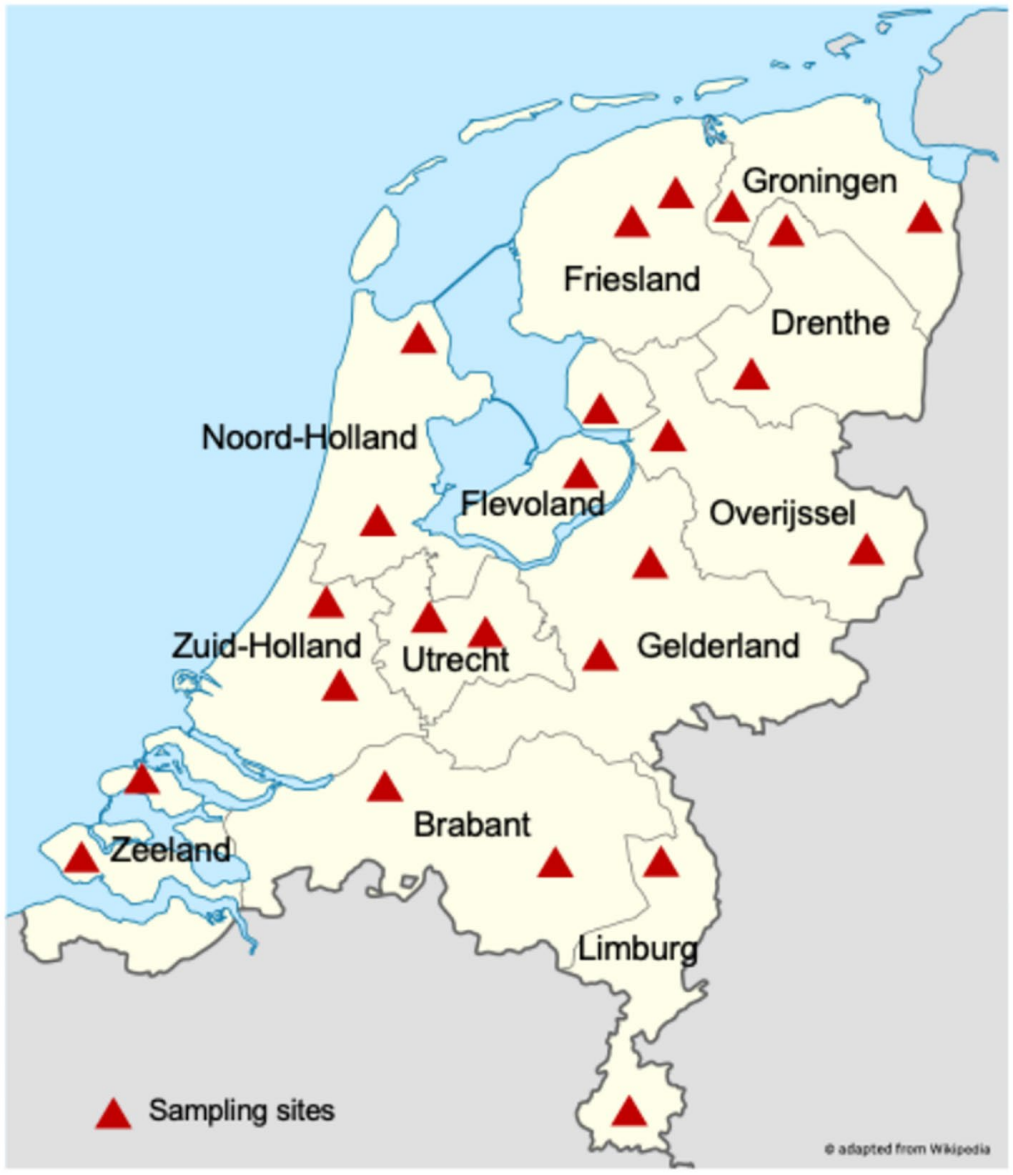
Sampling sites in the Netherlands. Two sheep farms were sampled in each province, with 10 sheep foot swab samples obtained per farm.

A total of 240 foot swab samples were collected from sheep farms across the Netherlands (Figure 1). In nine samples, the *pnpA* qPCR was inhibited, and these were discarded from the analysis. The remaining 147 samples were positive for *D. nodosus,* and 8 samples were *pnpA* qPCR-negative.

The multiplex qPCR identified at least one serogroup in 144 samples (98%) of 147 *pnpA*-positive samples, and serogroup cPCR detected a serogroup in 44 (28%) of the *pnpA-*positive samples (Table 4A). It was established that only samples with a *pnpA* Ct-value of 27 or lower could facilitate serogroup detection in samples using serogroup cPCR. Comparison of the numbers detected per serogroup by qPCR versus cPCR showed a significantly higher proportion of serogroup detection by qPCR for serogroups A, B, C, E, G, and H (*p* < 0.05). After Bonferroni correction, the significance only changed for serogroup C to non-significant (Table 4B). For serogroups D, F, and I, the proportion of serogroups detected by qPCR or cPCR was not significantly different, probably due to the low number of positive samples. For serogroup D, in five samples, this serogroup was detected by cPCR and not by qPCR (Supplementary Table 2).

**Table 4:** Metrics of the multiplex qPCR compared to cPCR for detection of *D. nodosus* serogroups in sheep foot swabs.

(A) number of samples with a serogroup detected by qPCR and cPCR				
		qPCR		
		+	−	Total
cPCR	+	44	**0**	44
	−	100	3	103
	Total	144	3	147

(B) serogroup detected by qPCR versus cPCR				
Serogroup	McNemar test statistics	*p*-value	*P* < 0.05	*P* < 0.005*
A	47.02	7.03E-12	Yes	Yes
B	56.02	7.18E-14	Yes	Yes
C	4.17	4.12E-02	Yes	**No**
D	0.00	1.00E+00	**No**	**No**
E	16.06	6.15E-05	Yes	Yes
F	3.20	7.36E-02	**No**	**No**
G	10.08	1.50E-03	Yes	Yes
H	6.13	1.33E-02	Yes	No
I	1.33	2.48E-01	**No**	**No**

(C)	Total number of positive samples		Proportion of positive samples		*P*-value	
Serogroup	cPCR +	qPCR +	cPCR +	qPCR +	(McNemar)*	
A	8	57	0.05	0.39	0.00000	**<0.001**
B	14	72	0.10	0.49	0.00000	**<0.001**
C	6	12	0.04	0.08	0.04123	<0.05
D	6	7	0.04	0.05	1.00000	1.000
E	0	19	0.00	0.13	0.00006	**<0.001**
F	14	19	0.10	0.13	0.07364	0.074
G	0	12	0.00	0.08	0.00150	**<0.005**
H	3	11	0.02	0.07	0.01333	<0.05
I	4	7	0.03	0.05	0.24821	0.248

qPCR = multiplex realtime PCR, cPCR = conventional PCR. A: Overall number of samples with at least one serogroup detected for both methods, B: Concordance between specific serogroups detected by both methods, and C: The proportion of samples positive for specific serogroups for both methods. * Bold values are significant, including Bonferroni correction (p <0.005).

The multiplex qPCR assay detected a single serogroup in 102 samples and multiple serogroups in 46 samples and could detect up to five different serogroups in a single foot swab. In contrast, cPCR identified eight samples with more than one serogroup, with up to three serogroups per sample. In a cross-table with detection of all serogroups per sample, it is shown that there is a linear distribution of serogroups detected per sample (Supplementary Table 3), and when all the serogroups that were detected were compared, qPCR detected statistically significant (*p* < 0.001, Wilcoxon signed-rank test) more serogroups per sample than cPCR.

**Figure 2:**
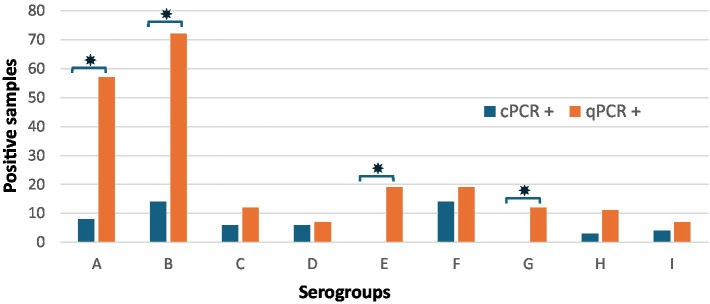
Total number of foot swab samples in which a serogroup was detected by cPCR and/or qPCR (*n* = 147). The bars show the number of samples positive for serogroups A to I, with the number of positive samples on the *Y*-axis and the serogroups on the *X*-axis. * Indicates a significant difference (*p* < 0.005).

The proportion of serogroups detected in a sample was significantly higher for serogroups A, B, E, and G in the qPCR (*p* < 0.001, Table 4C). Primarily, serogroups A (*n* = 58/147) and B (*n* = 73/147) were detected. Unlike cPCR, the qPCR identified serogroups E (*n* = 19/147) and G (*n* = 12/147) (Figure 2).

## Discussion

In this study, a multiplex qPCR was evaluated for accurate serogroup detection of *D. nodosus* in sheep foot swab samples that can support the development of flock-specific vaccination strategies (12, 29). Major advantages of targeted monovalent and bivalent footrot vaccines over multivalent vaccines have been that they provide longer-term prevention and control and have been found to be very effective in the eradication of endemic outbreaks (12, 20, 29). Designing an effective flock-specific vaccine strategy requires accurate serogroup identification, as targeting the wrong serogroups would nullify vaccine effectiveness (20).

The qPCR in this study detected the *D. nodosus* serogroup variation confined to the carboxy-terminal region of the relatively small fimbrial gene (*fimA*), which spans only 800 to 900 base pairs (8, 9). The nucleotide homology of the *fimA* gene varies between serogroups, ranging from 60 to 86% (6), which presented a challenge in designing serogroup-specific primers and probes. The nucleotide homology of the *fimA* gene varies between serogroups, ranging from 60 to 86% (6), which presented a challenge in designing serogroup-specific primers and probes, particularly for serogroups C and G, which show close genetic similarities. Nevertheless, the probe sequences for C and G were serogroup-specific, although minor mismatches were observed in the primer sequences. These mismatches are unlikely to significantly impact qPCR efficiency (30). Furthermore, the qPCR assay demonstrated high specificity based on both *in silico* analysis and qPCR cross-reaction analysis (Table 3). The sensitivity for detecting serogroups in foot swabs with qPCR was higher than the cPCR assay (Table 4). Three of 147 *pnpA*-positive clinical samples tested negative for all serogroups with the qPCR assay, representing a low false-negative rate. In comparison, culture is slower and less sensitive for detecting *D. nodosus* in samples with lower template concentrations. The detection of additional serogroups identified by qPCR and the relative quantification of *D. nodosus* DNA are advantages not possible with traditional PCR assays. Furthermore, the use of Ct-values in qPCR provides an added benefit by enabling estimation of the relative abundance of each serogroup within a sample and for monitoring the effect of interventions. These features make qPCR well suited for monitoring *D. nodosus* status on farms and integrating into footrot surveillance programs across European countries, where conventional serogroup PCRs are still commonly used (31–33).

Multiple studies in other countries have reported that sheep flocks commonly harbor more than one *D. nodosus* serogroup (10, 12, 25, 34, 35). Quantifying all present serogroups by qPCR enables identification of the most abundant ones, which is essential for the development of effective monovalent or bivalent vaccines and the design of a strategy for the sequential application of these vaccines. For low-abundance serogroups, it is important to determine whether they represent less virulent strains and whether they might outcompete dominant strains following vaccination with vaccines containing these latter, a risk that has been reported for other pathogens (36). In addition, the detection of multiple serogroups within a flock requires additional information about the virulence status of these serogroups. This information is crucial for deciding whether vaccination should target the two most abundant and/or virulent serogroups. It is important to note that the qPCR assays were designed for serogrouping and do not differentiate between virulent and benign strains, necessitating the use of an additional test to address this issue. Clinical diagnosis can be necessary if one or two serogroups are detected in a flock to check for virulence, and phenotypic microbiological tests, such as elastase and gelatin gel, or the qPCR test targeting *aprV2* and *aprB2* (37, 38), may be used to differentiate between virulent and benign strains, although phenotypic methods are not always reliable (37, 38). Further molecular investigations of virulence-associated genetic regions in *D. nodosus* could extend the use of PCR-based tests on virulence that can be combined with serotyping for more accurate diagnosis in targeted vaccination strategies (20).

The commonly present microbial populations in lesion samples (22, 39, 40) can challenge specific diagnoses of *D. nodosus*. In this study, we preceded the serogroup qPCR with the *pnpA* qPCR, which has been shown to be specific and sensitive for *D. nodosus,* to identify the presence of *D. nodosus* (27, 28). This enhances the diagnostic test procedure’s specificity, making it effective in clinical settings where mixed microbial populations are common (22, 39, 40). In *pnpA*-positive samples, the serogroup qPCR accurately distinguished serogroups in swabs with mixed infections and showed higher sensitivity for serogroup detection than the serogroup cPCRs. Only detection of serogroup D needs improvement by determining whether reduced sensitivity is due to sequence variability or sample-induced inhibition, as the multiplex qPCR identified fewer serogroup D detections in samples than cPCR. Further validation of the multiplex qPCR assays with additional sequencing of *D. nodosus* from different environments and flock conditions is recommended to ensure that sequence variability is adequately captured, thereby maintaining assay performance.

In conclusion, the developed multiplex qPCR for diagnosis of *D. nodosus* serogroups rapidly provides data on the relative abundance of each serogroup within samples. This serogroup-specific diagnosis is crucial for epidemiological studies and for tailoring vaccines to target the prevalent *D. nodosus* serogroups within sheep flocks (22, 39–41).

## Data Availability

The original contributions presented in the study are included in the article/Supplementary material, further inquiries can be directed to the corresponding author/s.
